# Workplace Challenges in Schizophrenia: Characteristics and Implications of long-term medical leave

**DOI:** 10.1192/j.eurpsy.2026.12124

**Published:** 2026-07-31

**Authors:** Y. Tebourbi, G. Bahri, Y. Yaich, G. Amri, M. Mersni, S. Chmengui, D. Brahim, M. Bani, R. Ghachem, N. Ladhari

**Affiliations:** 1 Occupational Medicine and Professional Pathologies Department, Charles Nicolle Hospital, Tunis; 2 Psychiatry Department B, Razi Hospital, Manouba, Tunisia

## Abstract

**Introduction:**

Employees with schizophrenia face significant challenges in maintaining continuous employment and often require extended medical leave periods. Understanding these leave patterns is essential for developing effective workplace support strategies and informing occupational health policies for this vulnerable population.

**Objectives:**

The aim of this study was to describe the patterns and characteristics of long-term medical leave among employees diagnosed with schizophrenia.

**Methods:**

A descriptive, retrospective study was conducted using medical records of patients with schizophrenia referred to the Occupational Health Department at Charles Nicolle University Hospital for medical leave assessment between 2022 and 2025.

**Results:**

A total of 40 cases were analyzed. Most participants were male (52.5%) with a median age of 44 years (range: 27-61) and a mean professional seniority of 13.4 ±7.6 years. The most represented occupations were laborers (27.5%), senior technicians (20%), and nurses (12.5%). The median duration of medical leave was 60 days (range: 10-90). The most common reason for leave was ill health (65%), followed by social reasons (17.5%) and professional reasons (17.5%). Regarding leave history, 45% were on their first leave, 32.5% were on their first extension, and 12.5% had multiple extensions. Overall, 95% of leaves were deemed justified, while 5% were considered fit to immediately resume their professional activity.

**Conclusions:**

Employees with schizophrenia often require medically justified periods of leave, highlighting ongoing difficulties in achieving long-term workplace integration. These findings underscore the need for supportive employment policies.

**Disclosure of Interest:**

None Declared

